# Practical Notes and Formulæ

**Published:** 1888-02

**Authors:** 


### PRACTICAL NOTES AND FORMULÆ.

**Asthma.**—Dr. T. L. Lallerstedt, of Panola, Ga., writes: I have been trying a new treatment for asthma. I send you the formula that your readers may report the result after a fair trial. I have come to believe that asthma and all allied diseases are caused by bacteria in the bronchial tubes, and anything tending to destroy them will give relief. The prescription No. 1, is:

℞. Piperin....................................grs. iv,
Oil sassafras................................gtts. xii,
Cinchonidia sulph..........................grs. xx.

M. Put into 8 capsules pulv. valerian, grs. xii. Dose: take a capsule every six hours until relief is obtained; and then if an attack came on at night take 1 capsule at 2 and 8 o'clock p. m.

No. 2. ℞. Iodine............................grs. xx,
Iodide of potass..........................ʒ ii,
Water........................................℥ i,
Let dissolve,
Add tr. nux vomica.........................℥ ii.

Dose: Fifteen drops, twenty minutes after each meal, in half glass of water. Shake well before taking.

Hope your readers will give this a trial.

**For Chronic Catarrh.**—(*Da Costa*)

℞. Potas. acetatis..............................℥ j,

Tinct. ferri chlor.............................f ℥ j,

Acid. acetic. dil...............................f ℥ ss,

Elixir simplicis...............................f ℥ j,

Aquæ, q. s. ad..................................f ℥ vj.

M. Sig.—Teaspoonful four times a day.

**For Stomatitis.**—The following gargle is recommended by Monin in this affection:

℞. Acidi borici....................................
Acidi salicylici..............................aa gr. iv,
Potassii chloratis..............................ʒ ij,
Glycerini.........................................℥ iss,
Essent. myrrhæ.................................gtt. xvi,
Aq. aurantii flor...............................℥ ixss.

M. Sig.—Use as a gargle.—*L'Union Medicale.*

**Nasal Catarrh—Folicular Pharyngitis.**—A writer in *Medical Brief* says: "The past two years I have been very successful in treating patients suffering with nasal catarrh and folicular pharyngitis. The treatment I use, will give for the benefit of readers of the *Brief*. For douche applied by means of catarrhal syrnge inserted back of soft palate, and readily finds exit through the nose. I use the following:

℞. Kennedy's Pinus Canadensis (dark)............ ʒ i,

Listerine.................................. ʒ i,

Glycerine.................................. ℥ ss,

Aquæ font.................................. ℥ ii.

M. Sig.—To be used by physicians twice a week.

And if there is much trouble in the throat use for gargle:

℞. Kennedy's pinus can (dark).................. ʒ iss,

Listerine.................................. ʒ ii,

Tinct. capsici............................. ʒ i,

Tannic acid................................ ʒ ss,

M. Sig.—Gargle in throat twice daily.

I have cured a great many cases by this routine treatment in from three to six months. The only trouble is to keep your patients from getting discouraged the first month, then they begin to feel marked relief.

By this mode of treatment you will cure chronic cases that have suffered for fifteen or twenty years.

**Salicylic Plaster Comp.**—(*Medical World*)

℞. Emplastri diachyli sim...........................
Emplastri saponati......................... aa℥ i,
Petrolati.................................. ʒ iii,
Acidi salicylici........................... ʒ i.

M. S.—Spread on muslin or other suitable fabric.

This plaster is yellowish gray, without offensive odor, and convenient for spreading. It is very useful in chronic eczema, old leg ulcers, etc. It was developed by Mr. Rutenik, and is adopted by the German hospital and dispeneary of New York.—*Ib.*

**Muriate of Ammonia.**—A writer in *American Med Journal* recommends the following as a specific for the delirium of typhoid fever:

℞. Hydrochlorate ammonia syr......... ℥ j, (60 gr. to the oz.)

Fl. ext. valerian.................. ʒ iv,

M. Sig.—Dose; one-half to 1 drachm in gum acacia, every two to four hours, according to condition of patient or urgency of case. I have seen patients recover their senses and arouse from stupor with the use of the above formulæ, when all hope of recovery had been abandoned.

**A most** valuable remedy for *functional impotence*, especially when accompanying hypochondriasis, is the chloride of gold and sodium.—*College and Clinical Record.*

